# Study of the Effect of Modified Aluminum Oxide Nanofibers on the Properties of PLA-Based Films

**DOI:** 10.3390/ma15176097

**Published:** 2022-09-02

**Authors:** Anna Sukhanova, Anatoly Boyandin, Natalya Ertiletskaya, Mikhail Simunin, Taisia Shalygina, Anton Voronin, Alexander Vasiliev, Ivan Nemtsev, Mikhail Volochaev, Svetlana Pyatina

**Affiliations:** 1 Scientific Laboratory “Smart Materials and Structures”, Reshetnev Siberian State University of Science and Technology, 31 Krasnoyarsky Rabochy Av., Krasnoyarsk 660037, Russia; 2 Institute of Biophysics, Federal Research Center “Krasnoyarsk Science Center SB RAS”, 50/50 Akademgorodok, Krasnoyarsk 660036, Russia; 3 Department of Molecular Electronics, Federal Research Center “Krasnoyarsk Science Center SB RAS”, 50 Akademgorodok, Krasnoyarsk 660036, Russia; 4 School of Engineering and Construction, Siberian Federal University, 82K Svobodnyi Av., Krasnoyarsk 660041, Russia; 5 Kirensky Institute of Physics, Federal Research Center “Krasnoyarsk Science Center SB RAS”, 50/38 Akademgorodok, Krasnoyarsk 660036, Russia; 6 Institute of Engineering Physics and Radio Electronics, Siberian Federal University, 79 Svobodnyi Av., Krasnoyarsk 660041, Russia; 7 Insitute of Fundamental Biology and Biotechnology, Siberian Federal University, 79 Svobodnyi Av., Krasnoyarsk 660041, Russia

**Keywords:** aluminum oxide nanofibers, percolation threshold, polylactide, film, composite, thermal and mechanical properties

## Abstract

To find out whether Al_2_O_3_ nanofiller is effective in improving the characteristics of polymer composites, composite polymer films based on biodegradable polylactide and epoxidized aluminum oxide nanofibers were obtained by solution casting. Surface morphology, mechanical and thermal properties of composites were studied by SEM, IR-Fourier spectroscopy, DSC and DMA. It was shown that, below and above the percolation threshold, the properties of the films differ significantly. The inclusion of alumina nanoparticles up to 0.2% leads to a plasticizing effect, a decrease in the crystallization temperature and the melting enthalpy and an increase in the tensile stress. An increase in the content of alumina nanoparticles in films above the percolation threshold (0.5%) leads to a decrease in the crystallinity of the films, an increase in stiffness and a drop in elasticity. Finding the percolation threshold of alumina nanoparticles in PLA films makes it possible to control their properties and create materials for various applications. The results of this study may have major significance for the commercial use of aluminum oxide nanofibers and can broaden the research field of composites.

## 1. Introduction

Among the total amount of plastic waste, single-use plastics (SUPs) (e.g., plastic bags, packages, gloves, etc.) account for around 40% of debris [1]. In this regard, the current situation requires a rational approach to the use of plastic products and finding alternative solutions to the problem of excessive plastic use. Switching to fully biodegradable polymers prone to degradation/decomposition under appropriate environmental conditions is a necessary strategy in this situation.

Polylactide (PLA) is one of the most well-known biodegradable polymers. PLA is obtained from renewable sources (corn, sugar beet, wheat, etc.) and widely used in medicine, packaging industry, textile and automotive industries [2]. However, the widespread use of PLA is still limited due to a number of drawbacks related to its processing and physical and mechanical properties. PLA products are highly fragile and possess low elongation at break (about 3%) and impact failure (~2.5 kJ/m^2^, according to Charpy impact test values). Moreover, PLA undergoes gradual crystallization and enzymatic hydrolysis throughout its shelf life [3]. To overcome these disadvantages, a number of ways have been proposed for modifying PLA, such as reinforcement, addition of plasticizers or blending with other polymers [4,5,6]. The inclusion of reinforcing materials in biopolymer matrix significantly improves tensile strength and toughness of the resulting composites [7,8]. Reinforcement by nanoparticles (NP) and nanofibers (NF) results in an increase in Young’s modulus, tensile strength and heat deflection temperature, a decrease in gas permeability, and improved biodegradability [7].

Among the natural compounds used as reinforcing nanomaterials for biopolymers the following groups are distinguished: (i) natural fillers, such as layered aluminosilicates of nano-/microparticles: halloysite, bentonite, montmorillonite, hydroxyapatite, calcium carbonate, etc. [7,9]; (ii) natural fibers, such as wood and vegetable fibers [10,11]; and (iii) naturally occurring antioxidant molecules (nAO), such as phenols, polyphenols, vitamins and carotenoids [12,13].

Recently, aluminum oxide nanofibers (AONF), which are obtained by growing from a melt in the presence of a non-wettable refractory component in the solid phase, have been of particular interest [14]. The length of the resulting fibers ranges from fractions of a micron to a centimeter, and the diameter of a single fiber ranges from 3 to 45 nm [15].

There are known examples of using AONF as a binding and reinforcing agent to improve mechanical properties and fire resistance of polymers, such as ethylene and propylene, polyamide, polyvinyl alcohol (PVA), polyvinylpyrrolidone (PVP), polyethylene oxide (PEO), polyacrylonitrile (PAN) [16,17,18,19,20,21].

Data on the obtaining and studying of such composites based on biodegradable polymers, in particular based on PLA with aluminum oxide, are quite limited. Only a few studies have demonstrated the effect of hydrated aluminum oxide (boehmite) on the values of stretching and a significant improvement in tensile strength (57%) and elongation at break with 3 wt.% boehmite loading [22]. In another study by Abdul Mujeeb, the addition of aluminum oxide and copper nanoparticles resulted in an increase in the glass transition temperature (53–57 °C) and melting point (146–153 °C) of composite samples, compared to pristine PLA [23].

Lule and Kim studied composite films with aluminum oxide nanoparticles (18 wt.%, 28 wt.% and 38 wt.%) and aluminum nitride (2 wt.%) obtained by solution casting. The thermal conductivity of the treated composite films with 40% filler loading reached 0.715 W∙m^−1^∙k^−1^, which was 150% higher compared to that of neat PLA [24].

It should be noted that the inherent specifications of composites are influenced by sufficient dispersion of the filler and interfacial interaction between the reinforcing agent and the polymer matrix. To find out whether Al_2_O_3_ nanofiller is effective in improving characteristics of polymer composites (PC), composite polymer films based on PLA and AONF as a filler were obtained by solution casting. The interaction of AONF with the polymer matrix and its effect on physical–mechanical and thermal properties of the biopolymer were studied.

## 2. Materials and Methods

### 2.1. Materials

A biodegradable polymer of lactic acid polylactide (PLA) with an average molecular weight (M_w_) of 80 kDa, a degree of crystallinity (C_x_) of 18%, and a melting point (T_m_) of 165 °C, purchased from Sigma Aldrich (St. Louis, MO, USA), was used to obtain composite films. AONF were obtained by a proprietary patented technique (Krasnoyarsk, Russia). Reagent grade chloroform (Ecos-1, Russia) and chloroform stabilized with ~50 ppm of amylene (Ecos-1, Russia) were used as solvents.

### 2.2. Synthesis of AONF

AONF were obtained from ENAW-1199 aluminum using the molten aluminum oxidation technology [14]. The structure of the resulting AONF was similar to that used as a filler for other polymers [25,26]. The obtained nanofibers were synthesized with a highly oriented texture and had an extremely high aspect ratio of length, in the range of centimeters at nanometer diameters. AONF were subsequently coated with epoxypropyl functional groups for better affinity with the PLA matrix. To this end, AONF were treated in a solution of 3-glycidyloxypropyltrimethoxysilane (98% Evonik, Wesseling, Germany) in toluene (99.8%, Sigma-Aldrich Chemie, Steinheim am Albuch, Germany). For this silanization, 15 g of alumina nanofibers was taken and dried at a temperature of 140 °C in a ShS-40-02 SPU drying oven for about 8 h in order to remove all adsorbed water from the surface, and at the same time to preserve hydroxyl groups in the surface structure. Then, the nanofibers were dispersed in toluene using an overhead stirrer based on the SGR-1 YHCHEM reactor, with a stirring speed of 1000 rpm for 90 min. Next, the suspension was heated to 80 °C with stirring at 600 rpm for 40 min. The silane was added to the obtaining dispersion in the amount of 7 g. After stirring the mixture for an hour, it was cooled, rinsed with toluene, and decanted. Toluene residues were removed by drying at a temperature of 120–140 °C in a drying oven for 8 h.

### 2.3. Obtaining Composite Films

Composite films based on PLA and AONF were obtained by casting of the polymer solution followed by solvent evaporation. Previously, AONF were treated with ultrasound (Sonicator 3000, Misonix, Farmingdale, NY, USA) in chloroform stabilized with ~50 ppm of amylene for 15 min. The resulting highly dispersed suspension was introduced into a 2% PLA solution in chloroform, so that mass concentrations of AONF accounted for 0.1%, 0.2%, 0.5% and 1% of the polymer mass, followed by ultrasonication for 2 min. The resulting blends were degassed in a desiccator, put into a degreased Petri dish and left in a dust-free drying cabinet at room temperature for a few days. The concentrations of aluminum oxide nanofibers in the obtained composite films were selected in a way that some of them were below the percolation threshold, and some above. The percolation threshold was calculated using the known ratios [27] with the assumption that the volume excluded by nanofibers is proportional to the volume of the compounded polymer. Knowing the basic physicochemical properties of AONF [15] and the aspect ratio of nanofibers close to γ = 50, the percolation threshold is estimated to be approximately 0.22%.

### 2.4. Methods

#### 2.4.1. Scanning Electron Microscopy (SEM) and Transmission Electron Microscopy (TEM)

The microstructure of the composite films’ cross-sections was studied using scanning electron microscopy (Hitachi S-5500, Tokyo, Japan) and transmission electron microscopy (Hitachi HT7700, Tokyo, Japan). For SEM, the pre-treated samples were coated with a thin conductive layer of platinum (at 10 mA, for 45 s) using an EM ACE200 low vacuum coating system (Leica, Teaneck, NJ, USA). For TEM, the samples were previously dehydrated with ethanol and acetone and impregnated with a mixture of epoxy resins and araldite in a 4:1 ratio. Impregnation and polymerization were performed as described in [28]. A Leica EM UC7 ultramicrotome was used to obtain ultrathin sections..

#### 2.4.2. X-ray Diffraction Analysis (XRDA) and FTIR Spectroscopy

X-ray structure analysis and determination of crystallinity of initial materials and composite films were performed by employing a D8 ADVANCE X-ray powder diffractometer equipped with a VANTEC fast linear detector, using CuKa radiation (Bruker, AXS, Karlsruhe, Germany).

The chemical structure of the samples was identified by Fourier transformed infrared spectroscopy (FTIR spectroscopy) combined with the attenuated total reflectance (ATR) technique, using a Nicolet iS10 spectrometer (Thermo Scientific, Walthamm, MA, USA) and ITX Smart prefix (Thermo Scientific, Walthamm, MA, USA) with a diamond crystal. The measurements were carried out with a spectral resolution of 4 cm^−1^ in the range of 4000–400 cm^−1^ and averaged over 32 scans. The obtained FTIR spectra were processed by OMNIC^TM^ Professional Software (v.9.2, Thermo Scientific, Walthamm, MA, USA), using advanced ATR correction.

#### 2.4.3. Differential Scanning Calorimetry (DSC)

Calorimetric measurements of the samples in the mode of temperature modulation of the heat flow were performed using a differential scanning calorimeter DSC25 (TA Instruments, New Castle, DE, USA) in standard aluminum crucibles in the nitrogen atmosphere at a 70 mL/min flow rate. The samples were heated in the temperature range from −10 to 195 °C at a 10 °C/min rate. Modulation of the heat flow was carried out by a sinusoid with a 60 s period and a ±1 °C amplitude. The first heating was carried out up to 190 °C at a 20 °C/min rate.

#### 2.4.4. Dynamic Mechanical Analysis (DMA)

A thermomechanical study of the viscoelastic properties of the samples was conducted using a dynamic mechanical analyzer Q800 (TA Instruments, USA). Measurement of storage modulus (*E’*) of a sample was carried out using a tensile clamp and a linear temperature scanning in the heating mode from 25 to 150 °C at a 3 °C/min rate. The frequency of dynamic loading of the sample was 1 Hz and the relative deformation was less than 0.1%. The samples were investigated in the form of plates with a size of 30 mm × 12.5 mm × 0.65 mm.

### 2.5. Statistical Data Processing

Statistical processing of the results was carried out according to standard methods using the Microsoft Excel 2010 software package for Windows 8. All of the experiments were carried out in triplicates. For the obtained data, the mean, the mean square deviation and the confidence interval were calculated. All calculations were carried out for the significance level α = 0.05.

## 3. Results

### 3.1. Characterization of Composite Films

**Figure 1:**
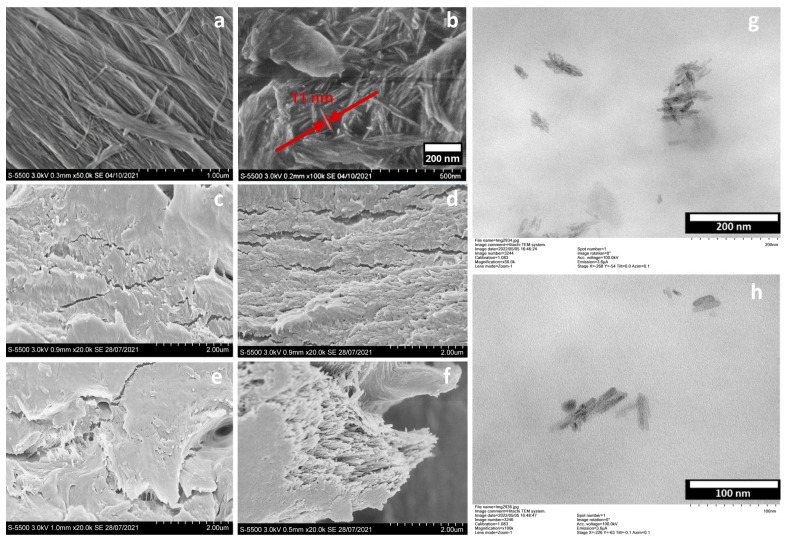
SEM images of AONF (**a**) and cross-sections of PLA (**b**) and PLA/AONF films with different content of AONF: (**c**)—0.1%, (**d**)—0.2%, (**e**)—0.5%, (**f**)—1%. TEM Images: (**g**,**h**)—PLA/AONF 1%.

A series of polymer composite films based on PLA and AONF as a reinforcing component was obtained and studied. SEM images of AONF and cross sections of the composite films are shown in Figure 1.

AONF was represented by tightly adjacent fibers and bundles ranging in size from tens to hundreds of micrometers (Figure 1a). The treatment of AONF with ultrasound before adding to the polymer solution led to the formation of fibers with the smallest length, from tens of nanometers to 0.2–0.5 μm, differing from the original length by 1–2 orders (Figure 1b). The structure of AONF practically did not change, and the fibers had a rod-like appearance with ragged edges. Sections of the PLA film and of all the composite films had similar morphology, with visible fractures and polymer lamellae of 30–85 nm in size. Since AONF are rather small in size and it is quite difficult to distinguish them in PLA/AONF composite films using SEM, we additionally applied TEM. Based on the microphotographs of 0.1% PLA/AONF films, it was found that AONF range in size from 30 to 80 nm, are prone to agglomeration and randomly arranged in the polymer matrix. The concentration of AONF below the percolation threshold (0.1%) leads to the emergence of free volume between AONF and polymer chains, leading to a change in the properties of the polymer (Figure 1h). 

### 3.2. FTIR

**Figure 2:**
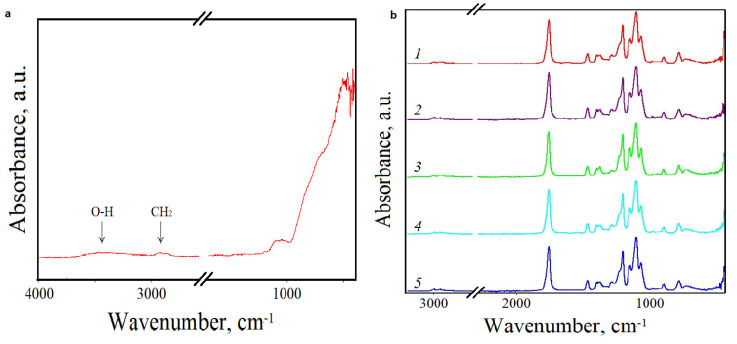
IR spectra of AONF (**a**), PLA (1) and PLA/AONF (**b**) composite films with AONF inclusion 0.1% (2), 0.2% (3), 0.5% (4) and 1% (5).

The IR spectra of the composite films are presented in Figure 2. The IR spectrum of unblended PLA is represented by distinctive bands corresponding to stretch vibrations of -CH in the CH_3_ group at 2944 cm^−1^ (symmetrical vibrations) and 2996 cm^−1^ (asymmetric vibrations). The peak at 1768 cm^−1^ corresponds to stretch vibrations of the carbonyl group (C=O). A number of bands in the range from 1091 cm^−1^ to 1211 cm^−1^ correspond to stretch vibrations of oxygen (in the C–O–C group). The IR spectrum of Al_2_O_3_ is specific for the hydroxyl group O-H and the methyl group CH_2_ peaks at 3463 cm^−1^ and 2921 cm^−1^. A peak near 1041 cm^−1^, which corresponds to H-O-H, and peaks around 100–500 cm^−1^, corresponding to the frequency of deformation vibrations and stretch vibrations of the Al-O bond, were also visible.

The inclusion of AONF in the polymer films did not lead to formation of new bonds and, as a result, new peaks did not occur in the IR spectra. Despite the fact that the epoxidized AONF groups are quite reactive, their interaction with the carboxyl group of the polymer or the terminal -COOH groups did not occur. The obtained results agree with recently published studies, which also did not reveal significant differences in PLA bands compared to PLA composites reinforced with metal oxides (MgO, ZnO and TiO_2_) [29,30,31,32].

### 3.3. XRDA 

**Figure 3:**
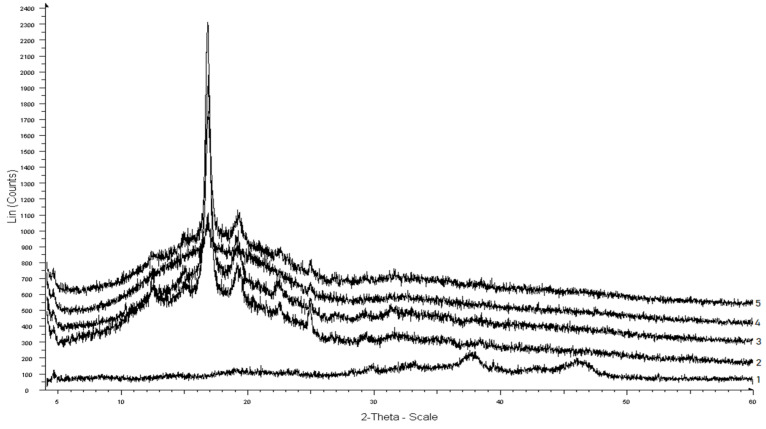
X-ray diffraction patterns of PLA (1) and PLA/AONF composite films with different AONF content: 2—0.1%, 3—0.2%, 4—0.5%, 5—1%.

PLA demonstrates various types of crystal modifications (α, β, γ and δ), depending on modes of synthesis and processing [33]. In this study, unblended PLA possessed the most common α-form, which crystallizes from a melt or solution, as confirmed by four diffraction peaks of 14.8°, 16.9°, 19.1°, and 22.5° (Figure 3). The X-ray diffraction pattern of AONF showed several diffraction peaks at 29°, 85°, 34°, 44°, 37.8°, and 46.5°, confirming the rhombohedral structure of γ-Al_2_O_3_, which is in agreement with the published data [34].

Compared with pure PLA, the X-ray images of PLA/AONF composite films were distinguished by an increase in the height intensity of diffraction peaks, which is due to the effect of AONF on the crystalline phase of the polymer, as has been shown previously [35]. In addition, the peaks of the crystal planes of PLA and AONF were in the same positions, which indicates the absence of any interaction between the components of the composites and agrees with the results of FTIR.

The crystallinity of composite films changed significantly compared to pure PLA. The crystallinity of PLA films was 18%, but when the AONF was added it dropped to 10%.

### 3.4. DSC

**Figure 4:**
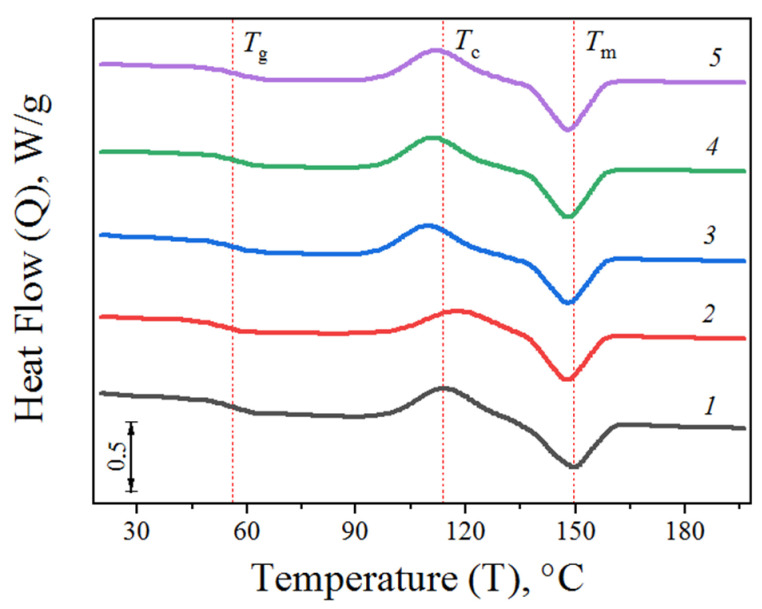
Results of DSC analysis of PLA (1) and PLA/AONF composite films with different AONF content: 2—0.1%, 3—0.2%, 4—0.5%, 5—1%.

According to the DSC analysis, the inclusion of AONF in PLA composite films led to a decrease in melting temperature from 149.5 °C (for unblended PLA) to 147.7 °C (Figure 4). Concurrently, changes in crystallization parameters were also observed, which did not correlate with the concentration of AONF. Composite films with the lowest AONF concentration (0.1 and 0.2 wt.%) were characterized by the greatest crystallization potential, due to the shift of T_c_ to the low-temperature range up to 113 °C. The sample of the composite film with 1% AONF inclusion, on the other hand, showed a shift in the crystallization temperature to the high-temperature range up to 121 °C (Table 1).

From the above results, it can be seen that the inclusion of AONF to PLA films affected both the amorphous and crystalline phases of the matrix. The absence of polymer–filler interaction at the interface boundary, the formation of extra free volume and the acquisition of additional conformational states in the chain segments of the amorphous phase led to an increase in their molecular mobility, which was confirmed by the changes in the crystallinity of the composites (Table 1).

This explains the decrease in the glass transition temperature T_g_ when the filler is introduced into the polymer matrix. In other words, a plasticizing effect of the filler was observed, which weakened when the filler inclusion was up to 1 wt.%. As a result of an increase in the molecular mobility of chain segments, the process of PLA crystals nucleation occurs at lower temperatures. However, it is possible to assume that the content of AONF did not affect the size of the formed crystallites, so the value of the melting point remained unchanged.

### 3.5. DMA

**Figure 5:**
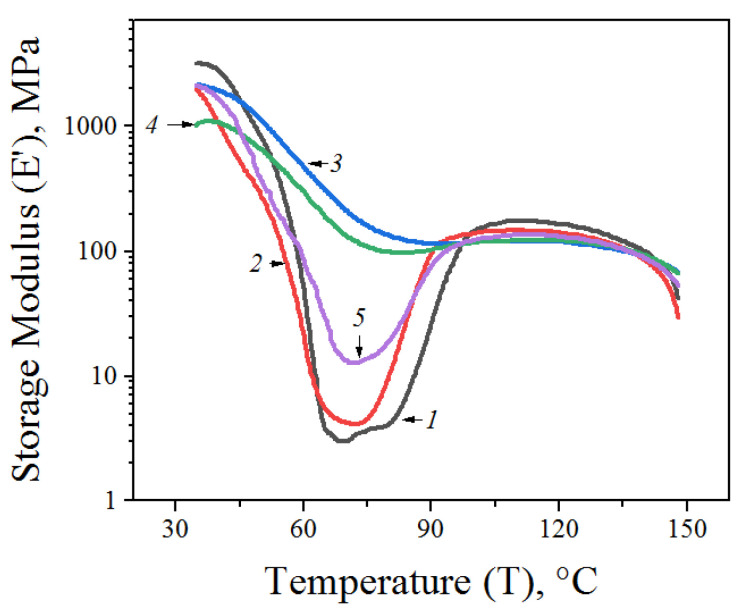
Storage moduli of PLA films (1) and PLA/AONF composite films with different AONF content: 2—0.1%, 3—0.2%, 4—0.5%, 5—1%.

According to the results of the DMA, a nonlinear dependence of *E’* on the content of AONF in a PLA composite was observed (Figure 5). At low filler content (0.1 wt% of AONF), *E’* had a value of 2500 MPa, which was 25% lower than the *E’* value for unblended PLA. A further increase in the AONF content up to 0.5 wt.% led to a gradual decrease in the *E’* value, down to 1100 MPa. However, at 1 wt.% content of AONF, an increase in *E’* up to 2200 MPa was observed. Probably, such effects were due to the fact that a high concentration of AONF filler contributed to the exhibition of the reinforcing effect of the nanofibers and, as a consequence, to an increase in the elasticity of the composite. 

**Figure 6:**
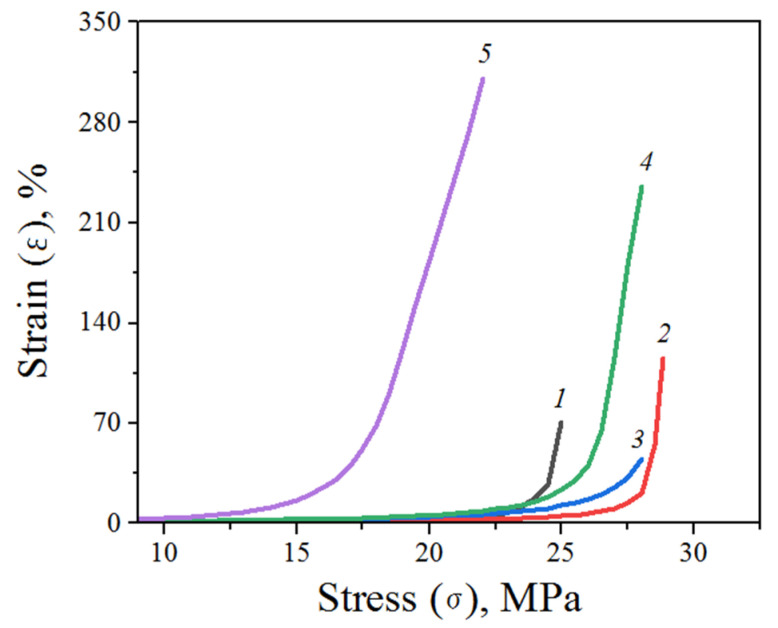
Stress–ultimate strain curves of PLA films (1) and PLA/AONF composite films with different AONF content: 2—0.1%, 3—0.2%, 4—0.5%, 5—1%.

Figure 6 shows the results of the DMA stretching test of PLA/AONF composite films. After addition of AONF, an increase in the relative strain was observed, indicating an increase in the elasticity of the composite films. However, a decrease in the strength of the films in the entire content range was also observed, indicated by a decrease in the value of tensile stress (Table 2). The values of mechanical properties of the nanocomposites are summarized in Table 2.

The data presented in Table 2 shows that the addition of AONF in a PLA matrix results in a change in mechanical properties. Summary data show that the addition of AONF leads to a decrease in Young’s modulus. Up to the percolation threshold, the tensile characteristics increase, while tensile stress decreases with an increase in the proportion of the additive. The maximum ultimate strength falls with a small amount of the additive, and decreases with an increase in its amount. Finally, the nanocomposite with the addition of AONF 0.2% stands out strongly in the data set; in this particular case, the tensile and strength characteristics deteriorate sharply for several identical samples.

## 4. Discussion

The behavior of a dispersed medium represented by nanofibers in an isotropic dispersion medium is described by the Onsager model [36]. In this study we rely on it, considering that PLA is a dispersion medium and aluminum oxide nanofibers represent a dispersed medium.

The surface charge of individual aluminum oxide nanofibers interacts with the PLA medium, and the nature of this interaction can be both loosening and coordinating. An important indication of the coordinating nature of the nanofiber–matrix interaction is the enthalpy of crystallization, the value of which is less in nanocomposite samples than in the pristine polymer. This results in nanofibers reducing crystallization energy as they serve as nucleation centers of PLA crystals [37]. As the number of nanofibers increases, the number of nucleation centers during cold crystallization increases as well. This should lead to a decrease in the average size of the grown crystals and a slowdown in the kinetics of their growth, due to an increase in “consumers” of the crystallized mass. The latter is indicated by an increase in the peak temperature and enthalpy of cold crystallization. With an increase in the content of aluminum oxide nanofibers, they begin to intersect with each other, forming intertwining. With a specific content of nanofibers, macroscopic clusters of nanofibers begin to form, which affect the behavior of the whole system. In this study, we observe a behavior similar to Raoult’s Law, but for low dimension of the additive concentrations. In particular, the growth of polymer crystals begins in different parts of such an intertwining and, as crystallization occurs, the inside growth proceeds from several competing centers simultaneously. This process leads to the need for diffusion of the consumable crystallization material into the cluster, i.e., when heated, the material will not have time to crystallize at the same rate as pristine PLA. We observed this in the nanocomposite with a 1% nanofiber content, where the peak temperature of cold crystallization is higher than in pristine PLA.

Notably, the values of the enthalpy of cold crystallization are close to the enthalpy of melting in the sample with 0.1% nanofiber content. It is obvious that the enthalpies of melting and cold crystallization are identical to phase transition temperatures. However, cold crystallization in PLA represents a transition from a highly elastic to a crystalline state, which is due to the mobility of polymer chains. This uniformity of the thermodynamic potential can be explained by the fact that the coordination of polymer chains around aluminum oxide nanofibers leads to its compaction at the interface and the formation of an intermediated free volume in the rest of the polymer medium. The formation of an intermediated free volume is confirmed by a decrease in the melting temperature, since the pristine PLA has more space for the mobility of its chains, resulting in increase in the number of microstates of the polymer belonging to the liquid phase, and, consequently, phase transition at lower temperatures becomes more probable. With an increase in the content of the filler, the volume of free PLA decreases, but this does not affect the proximity of the values of the enthalpies of melting and cold crystallization. We observed the greatest divergence for the sample with 0.2% aluminum oxide nanofiber content. This indicates a combination of the maximum of the mediated free volume, at which melting becomes easier, and the maximum of the nucleation centers, due to the combined growth, at which its rate slows down and a larger activation enthalpy of the process is required. This resulted in approaching the percolation threshold at this content of nanofibers.

The combined effect of the mediated free volume and the coordination of polymer chains around nanofibers also affects the temperature of transition to a highly elastic state. As the nanofibers do not form a full-fledged macroscopic cluster, the mediated free volume allows the nanocomposite to transition to a highly elastic state at a lower temperature than that of pristine PLA. However, immediately above the percolation threshold, the glass transition temperature returns to values close to pristine PLA. This is due to the fact that not only free PLA, but also that coordinated around aluminum oxide nanofibers, passes into a highly elastic state. A further increase in the content of nanofibers leads to the competition of already different macroscopic clusters that isolate in a separate phase, carrying away part of the polymer. This should lead to a weakening of the bonds between these clusters and an increase in the intermediated free volume. This is illustrated by a slight decrease in the glass transition temperature in composites with 1% aluminum oxide nanofiber content.

Thus, we envisage the behavior of the nanocomposite as follows. Aluminum oxide nanofibers interact with PLA by coordinating polymer chains around themselves. This interaction leads to loosening of the free polymer, since chains and their parts are partially removed from it, i.e., an indirect free volume is formed in the matrix due to the coordination of the polymer around the filler. As the amount of the filler increases, the volume of the free polymer decreases and that of the coordinated polymer increases. As the filler overcomes the percolation threshold, the free polymer is encased in a percolation network and a further increase in the filler content leads to the separation of macroscopic clusters of nanofibers into a separate phase.

The mechanical stretching test performed by DMA of PLA with different AONF content (Table 2) showed an increase in the ultimate strain of the material with an increase in the concentration of the additive. This is associated with a decrease in the Young’s modulus as the fraction of nanofibers in the polymer increases. It is clear that, with an increase in the number of nanofibers, the number of polymer-reinforcing sections increases. In turn, the drop in the Young’s modulus is associated with the loosening effect of the additive on the polymer structure. Thus, it can be concluded that AONF act as a plasticizing additive. The relationship between the ultimate strain and the ultimate stress is also remarkable. It transpired that the ultimate stress increases below the percolation threshold and decreases above it. This is quite possibly due to the fact that agglomerates of AONF begin to form in the substructure of the nanocomposite, which become centers of crack growth upon rupture. Similar results were observed in [25], where the ultimate stress of the nanocomposite also decreased above the percolation threshold. The addition of nanofibers leads to a significant increase in the ultimate strain. With 1% addition of AONF, the sample elongates more than four times. This effect can only be explained by the mutual disposition of polymer chains and nanofibers along the direction of the load. Of particular note is the result of testing PLA samples with 0.2% AONF, which was repeated on several samples. We observe a sharp decline in the elastic tensile strain, as well as in the ultimate strain and elastic tensile stress. We explain this behavior by the proximity of AONF concentration to the percolation threshold. If we assume that in the material one of the microstates has established, in which there is an integral substructure of nanofibers, then in cases where there are not so many such microstates as above the percolation threshold, even small external loads should lead to the destruction of such a substructure and internal restructuring within the nanocomposite. Obviously, in this case, the load can no longer lead to elastic deformation, and plastic deformations begin to occur in the material, i.e., the yield point occurs at a lower load. It is also worth noting that AONF, when they act as a reinforcing component, increase the cohesive energy. This is reflected in the ultimate stress value, which increases when nanofibers are added.

Such a representation is not only very much in line with the classical Onsager model, but also explains the changes in mechanical characteristics of the nanocomposites. Small introductions of nanofibers below and close to the percolation threshold increase the strength of the material, and as soon as macroscopic clusters of nanofibers begin to form, they tend to maintain their integrity and rupture along their conditional boundaries. This results in the tensile strength value dropping above the percolation threshold. On the other hand, it is the presence of macroscopic clusters of nanofibers that provides greater elongation at the moment of rupture.

## 5. Conclusions

A series of PLA/AONF composite films was obtained. It was found that, in 0.1% PLA/AONF films, AONF were prone to agglomeration and randomly arranged in the polymer matrix. The inclusion of AONF in the polymer films did not lead to formation of new chemical bonds. The crystallinity of PLA films was 18%, but it dropped to 10% after the AONF were added. The inclusion of AONF to PLA films affected both the amorphous and crystalline phases of the matrix, as demonstrated in a decrease in melting temperature from 149.5 °C to 147.7 °C and in the shift of T_c_ from 116.1 to 113 °C. The concentration of AONF below the percolation threshold (0.1%) leads to the emergence of free volume between AONF and polymer chains, leading to a change in the properties of the polymer. It was shown that tensile strain and Young’s modulus values of AONF-reinforced films vary depending on the proximity of percolation threshold, and with an increase in the AONF content up to 1%, the tensile strain increases from 0.92 to 1.16%, but the elasticity drops from 1.17 to 0.45 GPa. PLA can be reinforced with no more than 0.2% of AONF by solution casting, followed by solvent evaporation to obtain a slight plasticizing effect. The addition of 0.1% AONF leads to an increase in the ultimate strength and elongation of the polymer by 15%, while the elastic characteristics of the polymer nanocomposite do not change significantly. The addition of AONF generally leads to an increase in the extensibility of the material and the ultimate stretch before rupture is at its maximum at 1% addition of AONF, and is equal to 339%, i.e., three times higher than the pure PLA sample. Tensile strain also increases, but not on the same scale, from 0.92% in pure PLA to 1.16% in a nanocomposite with 1% AONF. The findings of this research may have significant implications for the industrial application of aluminum oxide nanofibers and may contribute to the field of composites research.

## Figures and Tables

**Table 1:** Thermal properties of stock material (PLA) and PLA/AONF composite films.

Sample	T_m_, °C	H_c_, J/g	T_c_, °C	H_m_, J/g	T_g_, °C
PLA	149.5	29	116.1	23	56.1
PLA/AONF 0.1%	147.5	22	112.7	22	53.7
PLA/AONF 0.2%	147.7	24	113.4	21	56.4
PLA/AONF 0.5%	147.7	25	115.6	25	57.0
PLA/AONF 1%	147.7	25	121.4	25	55.8

**Table 2:** Mechanical properties of stock material (PLA) and PLA/AONF composite films.

Sample	Tensile Strain, %	Ultimate Strain, %	Tensile Stress, MPa	Ultimate Stress, MPa	Young’s Modulus, GPa
PLA	0.92	100.2	10.80	25.1	1.17
PLA/AONF 0.1%	0.93	115.4	10.77	28.8	1.16
PLA/AONF 0.2%	0.63	59.0	6.00	28.3	0.95
PLA/AONF 0.5%	1.11	235.0	8.03	28.0	0.72
PLA/AONF 1%	1.16	339.0	5.24	23.3	0.45

## Data Availability

Not applicable.

## References

[1] Doun M., Morris J. (2019). Plastic & Climate: The Hidden Costs of a Plastic Planet. www.ciel.org/wp-content/uploads/2019/05/Plastic-and-Climate-FINAL-2019.pdf.

[2] Balla E., Daniilidis V., Karlioti G., Kalamas T., Stefanidou M., Bikiaris N.D., Vlachopoulos A., Koumentakou I., Bikiaris D.N. (2021). Poly (lactic Acid): A Versatile Biobased Polymer for the Future with Multifunctional Properties—From Monomer Synthesis, Polymerization Techniques and Molecular Weight Increase to PLA Applications. Polymers.

[3] Domb A.J., Kumar N. (2011). Biodegradable Polymers in Clinical Use and Clinical Development.

[4] Greco A., Ferrari F. (2020). Thermal behavior of PLA plasticized by commercial and cardanol-derived plasticizers and the effect on the mechanical properties. J. Therm. Anal. Calorim..

[5] Vorawongsagul S., Pratumpong P., Pechyen C. (2021). Preparation and foaming behavior of poly (lactic acid)/poly (butylene succinate)/cellulose fiber composite for hot cups packaging application. Food Packag. Shelf Life.

[6] Rizal S., Olaiya F.G., Saharudin N.I., Abdullah C.K., NG O., Haafiz M.K.M., Khalil H. P. S. A. (2021). Isolation of Textile Waste Cellulose Nanofibrillated Fibre Reinforced in Polylactic Acid-Chitin Biodegradable Composite for Green Packaging Application. Polymers.

[7] Ilyas R.A., Zuhri M.Y.M., Aisyah H.A., Asyraf M.R.M., Hassan S.A., Zainudin E.S., Sapuan S.M., Sharma S., Bangar S.P., Jumaidin R. (2022). Natural Fiber-Reinforced Polylactic Acid, Polylactic Acid Blends and Their Composites for Advanced Applications. Polymers.

[8] Halimatul M.J., Sapuan S.M., Jawaid M., Ishak M.R., Ilyas R.A. (2019). Effect of sago starch and plasticizer content on the properties of thermoplastic films: Mechanical testing and cyclic soaking-drying. Polimery.

[9] Oksiuta Z., Jalbrzykowski M., Mystkowska J., Romanczuk E., Osiecki T. (2020). Mechanical and Thermal Properties of Polylactide (PLA) Composites Modified with Mg, Fe, and Polyethylene (PE) Additives. Polymers.

[10] Singh A.A., Genovese M.E. (2021). Green and Sustainable Packaging Materials Using Thermoplastic Starch. Sustainable Food Packaging Technology.

[11] Kasa S.N., Omar M.F., Abdullah M.M.A.B., Ismail I.N., Ting S.S., Vizureanu P. (2017). Effect of Unmodified and Modified Nanocrystalline Cellulose Reinforced Polylactic Acid (PLA) Polymer Prepared by Solvent Casting Method Morphology, mechanical and thermal properties. Mater. Plast..

[12] Dintcheva N.T., D’Anna F. (2019). Anti-/Pro-Oxidant Behavior of Naturally Occurring Molecules in Polymers and Biopolymers: A Brief Review. ACS Sustain. Chem. Eng..

[13] Kirschweng B., Tátraaljai D., Földes E., Pukánszky B. (2017). Natural antioxidants as stabilizers for polymers. Polym. Degrad. Stab..

[14] Kutuzov M. (2013). Method and System for Alumina Nanofibers Synthesis from Molten Aluminum. ANF Technology Limited, Assignee..

[15] Simunin M.M., Voronin A.S., Fadeev Y.V., Mikhlin Y.L., Lizunov D.A., Samoilo A.S., Chirkov D.Y., Voronina S.Y., Khartov S.V. (2021). Features of Functionalization of the Surface of Alumina Nanofibers by Hydrolysis of Organosilanes on Surface Hydroxyl Groups. Polymers.

[16] Bravaya N.M., Saratovskikh S.L., Panin A.N., Faingol’d E.E., Zharkov I.V., Babkina O.N., Lobanov M.V. (2019). Influence of silane coupling agent on the synthesis and properties of nanocomposites obtained via in situ catalytic copolymerization of ethylene and propylene in the presence of modified Nafen™ Al2O3 nanofibers. Polymer.

[17] Wu H., Krifa M., Koo J.H. Functionalized Nafen™ alumina nanofiber reinforced Polyamide 6 nanocomposites: Mechanical, thermal and flame retardant properties. Proceedings of the SAMPE 2015.

[18] Panda P.K., Ramakrishna S. (2007). Electrospinning of alumina nanofibers using different precursors. J. Mater. Sci..

[19] Bravaya N.M., Galiullin A.B.N., Saratovskikh S.L., Panin A.N., Faingol’D E.E., Vasil’Ev S.G., Bubnova M.L., Volkov V.I. (2016). Synthesis and properties of hybrid materials obtained byin situcopolymerization of ethylene and propylene in the presence of Al_2_O_3_nanofibers (NafenTM) on catalytic systemrac-Et(2-MeInd)_2_ZrMe_2_/isobutylalumoxane. J. Appl. Polym. Sci..

[20] Rodaev V.V., Zhigachev A.O., Golovin Y.I. (2017). Fabrication and characterization of electrospun ZrO_2_/Al_2_O_3_ nanofibers. Ceram. Int..

[21] Sahalie N.A., Wondimkun Z.T., Su W.-N., Weret M.A., Fenta F.W., Berhe G.B., Huang C.-J., Hsu Y.-C., Hwang B.J. (2020). Multifunctional Properties of Al_2_O_3_/Polyacrylonitrile Composite Coating on Cu to Suppress Dendritic Growth in Anode-Free Li-Metal Battery. ACS Appl. Energy Mater..

[22] Das K., Ray S.S., Chapple S., Wesley-Smith J. (2013). Mechanical, Thermal, and Fire Properties of Biodegradable Polylactide/Boehmite Alumina Composites. Ind. Eng. Chem. Res..

[23] Mujeeb A., Lobo A.G., Antony A.J., Ramis M.K. (2017). An Experimental Study on the Thermal Properties and Electrical Properties of Polylactide Doped with Nano Aluminium Oxide and Nano Cupric Oxide. INAE Lett..

[24] Lule Z., Kim J. (2019). Thermally conductive and highly rigid polylactic acid (PLA) hybrid composite filled with surface treated alumina/nano-sized aluminum nitride. Compos. Part A Appl. Sci. Manuf..

[25] Kuular A.A., Simunin M.M., Bermeshev T.V., Voronin A.S., Dobrosmyslov S.S., Fadeev Y.V., Molokeev M.S., Volochaev M.N., Khartov S.V. (2020). The Influence of Alumina Nanofibers on the Physical and Mechanical Properties of Mineral-Filled Polyethylene: An Experimental Study. Tech. Phys. Lett..

[26] Kuular A.A., Voronin A.S., Markevich I.A., Bermeshev T.V., Simunin M.M. (2020). Mechanical properties UHMWPE/alumina nanofibers nanocomposite. J. Phys. Conf. Ser..

[27] Balberg I., Binenbaum N., Wagner N. (1984). Percolation Thresholds in the Three-Dimensional Sticks System. Phys. Rev. Lett..

[28] Gayer G. (1974). Electronic Histochemistry.

[29] Swarup K., Shukla M. (2018). Biofilms of polylactic acid, reinforced with nano-magnesium oxide, for food packaging. Intern. J. Biol. Macromol..

[30] Ahmed J., Arfat Y.A., Castro-Aguirre E., Auras R. (2016). Mechanical, structural and thermal properties of polylactide nanocomposite films reinforced with Ag–Cu and ZnO. Intern. J. Biol. Macromol..

[31] Ghozali M., Fahmiati S., Triwulandari E., Restu W.K., Farhan D., Wulansari M., Fatriasari W. (2020). PLA/metal oxide biocomposites for antimicrobial packaging application. Polym. Plast. Technol. Eng..

[32] Yakdoumi F.Z., Hadj-Hamou A.S. (2020). Effectiveness assessment of TiO_2_-Al_2_O_3_ nano-mixture as a filler material for improvement of packaging performance of PLA nanocomposite films. J. Polym. Eng..

[33] Jiang L., Shen T., Xu P., Zhao X., Li X., Dong W., Chen M. (2016). Crystallization modification of poly(lactide) by using nucleating agents and stereocomplexation. E-Polymers.

[34] Luo Y., Wang X., Wang C., Hu Y., Liu M. (2018). Alpha-to-gamma reverse phase transformation of molten removal in alumina ceramics laser processing. Ceram. Int..

[35] Buzarovska A. (2013). PLA Nanocomposites with Functionalized TiO_2_Nanoparticles. Polym. Plast. Technol. Eng..

[36] Onsager L. (1949). The effects of shape on the interaction of colloidal particles. Ann. N. Y. Acad. Sci..

[37] Nomai J., Suksut B., Schlarb A.K. (2015). Crystallization Behavior of Poly (lactic acid)/Titanium Dioxide Nanocomposites. KMUTNB Int. J. Appl. Sci. Technol..

